# Disseminated herpes zoster encephalitis induced by low-dose corticosteroids

**DOI:** 10.1016/j.jdcr.2025.06.030

**Published:** 2025-06-30

**Authors:** Natalie Soliman, Britney Le, Kelly M. Kimball, Dawn Merritt

**Affiliations:** a Ohio University Heritage College of Osteopathic Medicine, Cleveland/Dublin, Ohio; b Department of Dermatology, Riverside Methodist Hospital, Columbus, Ohio; c Oakview Dermatology, Athens, Ohio

**Keywords:** corticosteroid treatment, herpes zoster, immunosuppression

## Introduction

Herpes zoster (HZ) is caused by reactivation of the varicella-zoster virus (VZV) and typically presents as a unilateral, vesicular, painful rash with a dermatomal distribution. Immunocompromised patients are especially susceptible to VZV reactivation. In rare cases, the infection can progress to encephalitis, pneumonia, hepatitis, retinal necrosis causing blindness, and cerebral ataxia, among others.^1^ Disseminated herpes zoster (DHZ) is characterized by widespread vesicular lesions beyond the primary dermatome, often with the involvement of other organs, and is predominantly observed in immunocompromised individuals.

Corticosteroids have been used since the 1950s for a range of illnesses, including inflammatory and arthritic disorders, as well as in palliative care. Although effective, they are known to suppress immune function, predisposing to infections, including viral reactivations. High-dose corticosteroids are a well-established risk factor for DHZ, but the threshold for clinically significant immunosuppression remains uncertain. This report describes a rare case of disseminated HZ encephalitis occurring in an elderly patient on a low-dose prednisone regimen of 5 mg daily, possibly challenging our view of what qualifies as immunosuppression.

## Case report

**Fig 1:**
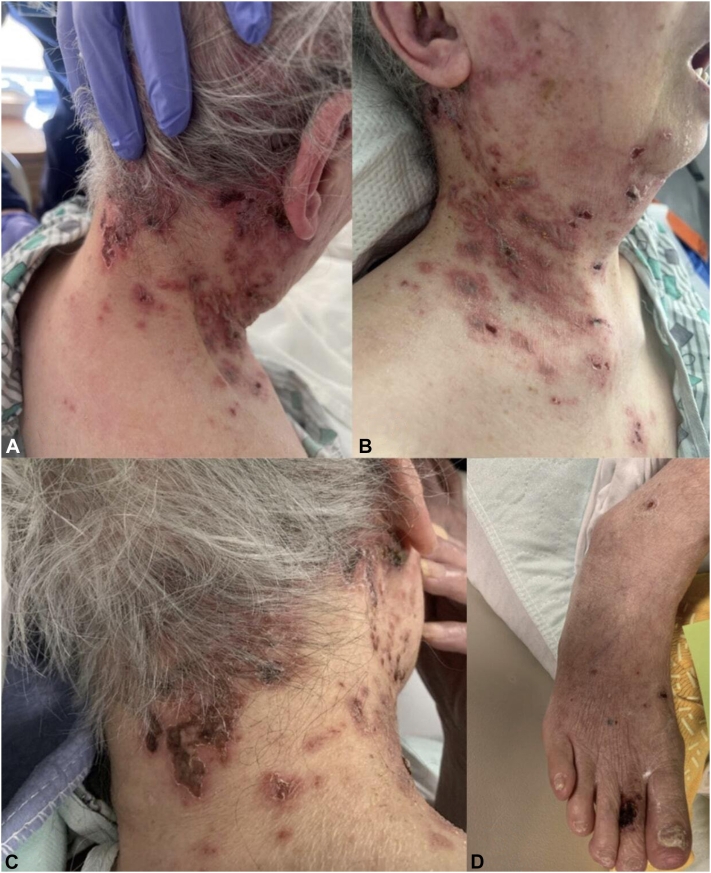
Clinical presentation of the patient with DHZ. **A-C,** Hemorrhagic vesicles and bullae on an erythematous base and can be noted in the cervical and postauricular region and involving the posterior and right third to fifth cervical (C3-C5) dermatomes. **D,** Involvement of the skin of the dorsal right foot and ankle. *DHZ*, Disseminated herpes zoster.

An 89-year-old woman presented to the emergency department for sudden-onset altered mental status and inability to walk or talk. She was reportedly fully independent at baseline. Due to her symptoms, a comprehensive stroke workup was completed (computed tomography head and neck) and showed no acute findings. On the first day of admission, a nonpainful, nonpruritic, bullous rash developed on her right occipital scalp, face, and neck which continued to spread to her bilateral lower extremities (Fig 1). There were no mucosal lesions. Due to the concern for bullous pemphigoid by the primary team, dermatology was consulted. She had received her annual influenza and COVID-19 vaccines but had not received the shingles vaccine. Her medication history included prednisone 2.5 mg twice daily, newly prescribed within the prior 2 months for the pain associated with degenerative disc disease. On physical examination, the patient was afebrile, normotensive, and nontachycardic. Labs showed a normal complete blood count, mild hyponatremia, and hyperglycemia. The patient was not diabetic, and her blood glucose was 144 on admission. No recent hemoglobin A1c was available. Autoimmune blistering diseases were ruled out with negative desmoglein 1 and 3 and bullous pemphigoid antigens. VZV/herpes simplex virus polymerase chain reaction swab was taken from a bullae on the neck. VZV polymerase chain reaction was found to be positive and the patient was promptly initiated on intravenous acyclovir 10 mg/kg every 8 hours. Due to the mental status change in the face of a positive VZV swab, a lumbar puncture was performed revealing elevated glucose, protein, red blood cell counts, white blood cell counts, and lymphocytes, with cerebrospinal fluid polymerase chain reaction confirming central nervous system VZV infection. Over the next 48 hours, the patient’s mental status rapidly improved. The final diagnosis was disseminated HZ encephalitis immunosuppression secondary to steroids.

## Discussion

Although most cases of HZ infection are self-limited, systemic corticosteroids and other immunosuppressive drugs can worsen the severity of the infection. To our knowledge, this is the first case report to document significant immunosuppression resulting in DHZ with a cumulative corticosteroid dose of 5 mg daily. While higher-dose corticosteroids are well-documented for VZV reactivation, our case shows that even minimal dosage can have profound immunosuppressive effects, prompting the reconsideration of what it means to be immunosuppressed from oral corticosteroids. Current literature on corticosteroid-induced DHZ involves patients receiving moderate to high doses, often in the setting of chronic inflammatory conditions or organ transplantation. A brief literature review of previously reported cases shows that systemic corticosteroid administration ranging from 7.5 mg to 20 mg daily (Table I) has contributed to the development of HZ. Our case demonstrates that doses as low as 5 mg daily can be sufficient to result in immunosuppression, resulting in VZV reactivation with dissemination and central nervous system involvement.

**Table I:** Previously reported cases of DHZ induced by immunosuppression from low-dose corticosteroids^2^, ^3^, ^4^, ^5^, ^6^, ^7^, ^8^

Author (name et al, year) and reference number	Age (y)	Biological sex	Comorbidity	Corticosteroid dose	Site of cutaneous herpes zoster lesion	Affected organs	Antiviral treatment	Outcome/ evolution
Zamora et al, 2019^2^	Mean 36.75 (±1.35)	93.8% female	Systemic lupus erythematosus	Mean 18.62 mg/d prednisone	Localized HZ in 97%; disseminated in 3%	2 patients had disseminated disease	Not reported	4 patients had >1 episode; HZ associated with high-dose steroids
Ferreira et al, 2016^3^	Under 18	89% female	Systemic lupus erythematosus	Median 20 mg/d prednisone (range 3-80 mg/d)	Thoracoabdominal (54%), limbs (22%), facial, back/lumbar, buttocks, cervical, genital	Not specified	IV or oral acyclovir	61% hospitalized; 13% had bacterial coinfections; 5% had postherpetic neuralgia; no deaths
Pappas et al, 2015^4^	Mean: 58.14 ± 13.48	76.2% female	Rheumatoid arthritis	7.5 mg/d	Not specified	Not specified	Not reported	729 HZ cases in 95,287 person-years; risk ↑ with age and steroids
Dubey & MacFadden, 2019^5^	70	Male	Rheumatoid arthritis, chronic obstructive pulmonary disease, congestive heart failure, coronary artery disease, atrial flutter, hypertension	10 mg/d	Started on forehead, progressed to entire body	Disseminated: lungs, colon, multi-organ failure	IV acyclovir	Death on day 5 due to multi-organ failure following disseminated vaccine-strain VZV infection after live vaccine
Ramachandran et al, 2020^6^	14	Female	Asthma	20 mg/d	Lower extremity (right knee)	Meninges	IV acyclovir and valacyclovir	Full recovery; confirmed vaccine-strain VZV meningitis in twice-immunized immunocompetent adolescent
Fujisato et al, 2018^7^	69	Male	Interstitial lung disease, type 2 diabetes mellitus, chronic kidney disease	IV methylprednisolone 1000 mg × 3 d, then prednisolone 60 → tapered to 40 mg/d	Genitals, left thigh, abdomen (disseminated, nondermatomal)	Meningoencephalitis	IV acyclovir	Fatal outcome due to aspiration pneumonia after DHZ-related meningoencephalitis
Timotijevic et al, 2024^8^	69	Female	Multiple myeloma	40 mg/d	Diffuse: back, abdomen, arms, legs, vulva	Lungs (pneumonia)	IV acyclovir	Rapid deterioration, acute respiratory failure, transitioned to comfort care; fatal outcome

*DHZ*, Disseminated herpes zoster; *Hz*, herpes zoster; *IV*, intravenous; *VZV*, varicella zoster virus.

Herein, we describe how seemingly low doses of systemic corticosteroids can predispose patients to varicella reactivation and, in some cases, dissemination. Although these outcomes may be uncommon, they warrant consideration, especially in the elderly. Health care providers must remain judicious when prescribing systemic corticosteroids, regardless of dosage, and they should closely monitor patients for potential complications, including VZV. This is particularly applicable to geriatric and immunocompromised populations. The patient in our case had been vaccinated for both influenza and COVID-19 but had no documented HZ vaccination. This underscores the importance of routine health maintenance and age-appropriate vaccination in the elderly, as this patient’s elderly age may alone have accounted for her risk of DHZ. Our case expands the understanding of corticosteroid-induced immunosuppression and its potential consequences. Nonetheless, the level at which systemic corticosteroids cause significant immunosuppression remains unknown and should be further investigated, especially in the geriatric and immunocompromised population.

## Conflicts of interest

None disclosed.
